# Reproductive factors and the risk of triple-negative breast cancer in white women and African-American women: a pooled analysis

**DOI:** 10.1186/s13058-016-0799-9

**Published:** 2017-01-13

**Authors:** Huiyan Ma, Giske Ursin, Xinxin Xu, Eunjung Lee, Kayo Togawa, Lei Duan, Yani Lu, Kathleen E. Malone, Polly A. Marchbanks, Jill A. McDonald, Michael S. Simon, Suzanne G. Folger, Jane Sullivan-Halley, Dennis M. Deapen, Michael F. Press, Leslie Bernstein

**Affiliations:** 1 Department of Population Sciences, Beckman Research Institute, 1500 East Duarte Rd. Duarte, City of Hope, CA 91010 USA; 2 Cancer Registry of Norway, Oslo, Norway; 3 Department of Nutrition, Institute of Basic Medical Sciences, University of Oslo, Oslo, Norway; 4 Department of Preventive Medicine, Keck School of Medicine, University of Southern California, Los Angeles, CA 90033 USA; 5 Section of Environment and Radiation, International Agency for Research on Cancer, Lyon, France; 6 School of Social Work, University of Southern California, Los Angeles, CA 90033 USA; 7 Division of Public Health Sciences, Fred Hutchinson Cancer Research Center, Seattle, WA 98109 USA; 8 Division of Reproductive Health, Centers for Disease Control and Prevention, Atlanta, GA 30333 USA; 9 College of Health and Social Services, New Mexico State University, Las Cruces, NM 88003 USA; 10 Karmanos Cancer Institute, Department of Oncology, Wayne State University, Detroit, MI 48201 USA; 11 Pathology, Keck School of Medicine, University of Southern California, Los Angeles, CA 90033 USA

**Keywords:** Reproductive factors, Breastfeeding, Triple-negative breast cancer, Luminal A, White women, African-American women

## Abstract

**Background:**

Early age at menarche, nulliparity, late age at first completed pregnancy, and never having breastfed, are established breast cancer risk factors. However, among breast cancer subtypes, it remains unclear whether all of these are risk factors for triple-negative breast cancer (TNBC).

**Methods:**

We evaluated the associations of these reproductive factors with TNBC, in 2658 patients with breast cancer (including 554 with TNBC) and 2448 controls aged 20–64 years, who participated in one of the three population-based case-control studies: the Women’s Contraceptive and Reproductive Experiences Study, the Women’s Breast Carcinoma *in situ* Study, or the Women’s Learning the Influence of Family and Environment Study. We used multivariable polychotomous unconditional logistic regression methods to conduct case-control comparisons among breast cancer subtypes defined by estrogen receptor, progesterone receptor, and human epidermal growth factor receptor-2 expression status.

**Results:**

TNBC risk decreased with increasing duration of breastfeeding (*P*_trend_ = 0.006), but age at menarche, age at first completed pregnancy, and nulliparity were not associated with risk of TNBC. Parous women who breastfed for at least one year had a 31% lower risk of TNBC than parous women who had never breastfed (odds ratio, OR = 0.69; 95% confidence interval, CI = 0.50–0.96). The association between breastfeeding and risk of TNBC was modified by age and race. Parous African-American women aged 20–44 years who breastfed for 6 months or longer had an 82% lower risk of TNBC than their counterparts who had never breastfed (OR = 0.18, 95% CI = 0.07–0.46).

**Conclusions:**

Our data indicate that breastfeeding decreases the risk of TNBC, especially for younger African-American women.

## Background

Breast cancer occurs more frequently than any other type of cancer in women worldwide, with an estimated 1.4 million new cases annually [1]. Based on immunohistochemical analyses of estrogen receptor (ER), progesterone receptor (PR), and human epidermal growth factor receptor-2 (HER2), breast cancer has been classified into luminal A-like (ER-positive (ER+) and/or PR-positive (PR+), HER2-negative (HER2–)), luminal B-like (ER+ and/or PR+, HER2-positive (HER2+)), HER2-enriched (ER-negative (ER–)/PR-negative (PR–)/HER2+), and triple-negative breast cancer (TNBC; ER–/PR–/HER2–) [2–10].

The luminal A-like subtype is the most frequent subtype and accounts for 62–67% of invasive cases; TNBC is the second most common subtype and accounts for 10–25% of invasive cases [11, 12]. Compared with the luminal A-like subtype, TNBC is disproportionately more common in younger or premenopausal women, especially young African-American women [13–16]. TNBC is biologically more aggressive and has poorer prognosis than the luminal A-like subtype [7, 12, 17]. Moreover, unlike ER+ or HER2+ breast cancer, for which there are targeted therapies, including anti-estrogen and monocolonal antibody therapies, there are no targeted therapies for TNBC [12, 18]. Therefore, currently the only treatment option for TNBC is systemic chemotherapy [12, 18]. The overall 5-year survival rate in patients with TNBC is at least 10% lower than in women with the luminal A-like subtype [19]. These TNBC characteristics underscore the need to identify specific TNBC risk factors, which could provide critical clues for TNBC prevention strategies. However, the impact of established breast cancer risk factors, such as reproductive factors, on the risk of TNBC remains inconclusive, possibly because too few patients with TNBC have been included in the majority of published studies [6, 10, 15, 20–32]. Moreover, little is known about the impact of race and age on reproductive risk of TNBC.

Here, we evaluated the associations between TNBC and age at menarche, number of completed (longer than 26-week gestation) pregnancies, age at first completed pregnancy, and breastfeeding in an analysis pooling data from three case-control studies. We compared the risk estimates for TNBC with other ER/PR/HER2-defined specific subtypes, especially luminal A-like breast cancer. We also explored whether the associations between these factors and risk of TNBC differ by race (white, African-American) and age (<45, ≥45 years). Among our three source studies, one has published data on reproductive factors (including number of completed pregnancies, age at first completed pregnancy, and breastfeeding) and the risk of breast cancer by ER/PR/HER2 status [6]. Another has published data on hormone-related risk factors for breast cancer by ER/PR status [33] and has also published characteristics of TNBC in patients with and without a *BRCA1* mutation [34]. No data on breast cancer risk according to the expression of ER, PR, or HER2 have been published from the third study [35].

## Methods

### Study population and data collection

Eligible participants for this analysis were women who had previously participated in one of the three population-based case-control studies - the Women’s Contraceptive and Reproductive Experiences (CARE) Study [6], the Women’s Breast Carcinoma *in situ* (BCIS) Study [35], or the Women’s Learning the Influence of Family and Environment (LIFE) Study [33].

The Women’s CARE Study, which was supported by National Institute of Child Health and Human Development (NICHD), was a population-based, case-control study designed to examine risk factors for invasive breast cancer among USA-born white women and African-American women [36]. The age distribution and participant response rates by study site, case-control status, and race have been published [36]. The Women’s CARE Study selected a stratified (by age group) random sample of women aged 35–64 years who were newly diagnosed with histologically confirmed incident invasive breast cancer (International Classification of Diseases for Oncology (ICD-O) codes: C50.0–C50.9) between July 1994 and April 1998. African-American women were oversampled to maximize their numbers in the study, and white women were sampled to provide approximately equal numbers of women in each 5-year age category (from 35 to 64 years).

Control participants were women with no history of invasive or *in situ* breast cancer who were identified by random digit dialing from August 1994 through December 1998 and were frequency-matched to the expected distribution of patients with breast cancer in strata defined by 5-year age groups, race (white or African-American) and geographic region of residence [6]. The participants in the Women’s CARE Study involved in the analyses presented here are women from Los Angeles (LA) and Detroit, the two sites where tumor tissue samples were collected. Tissue collection, as part of the Women’s CARE Study, was supported by NICHD, as advised by the Women’s CARE Study Steering Committee [36]. The Women’s CARE Study recruited 1921 case participants (1072 white and 849 African-American women) and 2034 control participants (1161 white and 873 African-American women) from LA and Detroit. Of 1921 case participants, 1206 had ER/PR/HER2 status assessed in a centralized pathology laboratory at University of Southern California (USC).

The Women’s BCIS Study investigated risk factors for BCIS among USA-born white women and African-American women who resided in LA County [35]. Case participants were USA-born and English-speaking white women and African-American women ages 35–64 years, who were newly diagnosed with a first primary BCIS (ICD-O codes: C50.0–C50.9) between March 1995 and April 1998 (*n* = 567). The questionnaire developed for the Women’s CARE Study was used to interview women with BCIS, and both studies were conducted during the same time period by the same interviewers and with ER, PR and HER2 status determined in the same central laboratory using the same classifications (see subsequent text). No additional controls were recruited for the BCIS Study. LA control participants from the Women’s CARE Study were deemed eligible to be controls for the BCIS Study. For the analysis presented here, we excluded 37 case participants with lobular carcinoma *in situ* (LCIS, ICD-O morphology code: 8520) because LCIS is not included in the clinical definitions of *in situ* breast cancer [37]; thus, 530 case participants remained. ER/PR/HER2 status was assessed in 343 of these case participants, at a centralized pathology laboratory at USC.

The Women’s LIFE Study investigated genetic and epidemiologic risk factors for invasive breast cancer in USA-born white women and African-American women who resided in LA County [33, 38]. Case participants were women aged 20–49 years who were diagnosed with a first primary invasive breast cancer (ICD-O codes: C50.0–C50.9) between February 1998 and May 2003 and who resided in LA county (*n* = 1794). Of 1794 case participants, 1167 had ER/PR/HER2 status abstracted from pathology reports. Control participants were women ages 20–49 years who had no history of invasive or *in situ* breast cancer. Recruitment of control participants did not begin until 1 July 2000. Control participants were individually matched by race (white and African-American), age (within 5 years and ages 20–49 years), and neighborhood to the subset of case participants who were diagnosed between 1 July 2000 and 31 May 2003 (*n* = 444). The Women’s LIFE Study used an expanded version of the Women’s CARE Study questionnaire, which was modified to include additional risk factors (e.g., medical radiation exposure).

For all three studies, detailed information prior to the reference date on reproductive factors and covariates involved in this analysis was collected by trained staff who administered standardized, in-person interviews using structured questionnaires. The reference date for a case participant was the date of breast cancer diagnosis; the reference date for a control participant was the date on which she was identified by random digit dialing in the Women’s CARE Study, or the date of initial contact in the Women’s LIFE Study.

After pooling the data from three source studies, 2716 case participants with data on receptors and 2478 control participants were potentially eligible. We excluded 58 case participants and 30 control participants for whom information was missing on age at menarche (4 cases, 1 control), parity (4 cases, 6 controls), duration of oral contraceptive use (16 cases, 5 controls), education (9 cases, 1 control), body mass index (BMI, 15 cases, 12 controls), recreational physical activity (5 cases, 3 controls), smoking status (2 cases), and alcohol intake (3 cases, 2 controls). This resulted in 2658 case participants (the Women’s CARE Study: 1197, the Women’s BCIS Study: 342, the Women’s LIFE Study: 1119) and 2448 control participants (the Women’s CARE Study or the Women’s BCIS Study: 2011, the Women’s LIFE Study: 437) available for the current pooled analysis.

### Assessment of biomarkers

The ER/PR/HER2 status in breast tumors in the Women’s CARE Study and the Women’s BCIS Study was determined in a centralized pathology laboratory at USC using immunohistochemistry (IHC) methods [39, 40]. For ER and PR, at least 100 tumor cells were examined from each specimen and immunostaining of tumor cell nuclei ≥1% was considered positive [41]. HER2 expression was determined by IHC using the 10H8 monoclonal antibody [42, 43]. No (0) or weak (1+) membrane immunostaining was considered HER2–. Moderate (2+) or strong membrane immunostaining (3+) was considered HER2+, based on previous validation results from the same pathology laboratory [42]. In the Women’s LIFE Study, the information on ER/PR/HER2 status was abstracted from pathology reports collected through the Los Angeles Cancer Surveillance Program (LACSP), a member of the population-based California Cancer Registry and also sponsored by the National Cancer Institute’s SEER program [34].

The ER/PR/HER2 status abstracted from pathology reports for case participants in the Women's LIFE Study was assessed by many pathology laboratories. These laboratories might have used different methods or different cut-off points for positive receptor status, which could cause concerns about consistency with the biomarker data from the centralized pathology laboratory at USC. We previously validated the SEER registry ER/PR status data for 1048 Women’s CARE case participants in the centralized pathology laboratory at USC, which showed that the agreement between the centralized laboratory and SEER registry classification was substantial for both ER/PR (κ statistics: 0.70 and 0.60 for ER and PR, respectively), and that the associations between risk of ER/PR breast cancer subtypes and parity, age at first completed pregnancy, and breastfeeding were similar regardless of the source of information on ER/PR [44].

### Statistical analyses

We assessed the associations between TNBC (ER-/PR-/HER2-) or the other three subtypes of breast cancer defined by ER/PR/HER2 status (luminal A-like, ER+ and/or PR+ plus HER2–; luminal B-like, ER+ and/or PR+ plus HER2+; and HER2-enriched, ER–/PR–/HER2+), and the following factors: age at menarche, number of completed (longer than 26-week gestation) pregnancies, age at first completed pregnancy (defined for each woman as the age at which that pregnancy ended), and duration of breastfeeding. We estimated odd ratios (ORs) and corresponding 95% confidence intervals (95% CIs) using multivariable polychotomous unconditional logistic regression for case-control comparisons [45].

Tests for trend were conducted by fitting ordinal values corresponding to exposure categories and testing whether the slope coefficient differed from zero. We also conducted Wald chi-square tests for homogeneity of the associations with reproductive factors across breast cancer subtypes by fitting a multivariable polychotomous unconditional logistic regression model for dichotomous or ordinal variables.

We included the following factors, selected a priori, as potential confounders in all multivariable models: source study (the Women’s CARE Study or the Women’s BCIS Study, the Women’s LIFE Study), study site (LA, Detroit), race (white, African-American), education as a proxy for socioeconomic status (high school or lower level of education, technical school or some college education, college graduate), age (<40, 40–44, 45–49, 50–54, 55–59, 60–64 years), family history of breast cancer (first-degree (mother, sister, or daughter), no first-degree family history), BMI (<25, 25–29, ≥30 kg/m^2^), a variable combining menopausal status and hormone therapy use (premenopausal; postmenopausal: never used hormone therapy, ever used hormone therapy; unknown menopausal status), lifetime recreational physical activity (inactive, ≤2.2, 2.3–6.6, 6.7–15.1, ≥15.2 annual metabolic equivalents of energy expenditure (MET) hours/week), alcohol intake (never, former, current), cigarette smoking status (never, former, current), and oral contraceptive use (never, <1, 1–4, 5–9, ≥10 years).

We also included age at menarche (≤12, 13, ≥14) and number of completed pregnancies (never pregnant, 1, 2, ≥3, only non-completed pregnancy) as potential confounders when they were not the exposure of interest. When parity was the exposure of interest, we chose women who had never been pregnant as our reference group and treated women who had been pregnant but had never carried to completed pregnancy as a separate group that was excluded when testing for trend across categories of parity. Only parous women were included in analyses of age at first completed pregnancy and breastfeeding. Models for parous women were also mutually adjusted for number of completed pregnancies (1, 2, ≥3), age at first completed pregnancy (≤20, 21–24, 25–29, ≥30 years), and duration of breastfeeding (never, <6, 6–11, ≥12 months).

Using two major subtypes - TNBC and luminal A-like breast cancer, we explored whether any associations of reproductive factors differed by race (white women or African-American women) or age group (younger (<45 years) or older (≥45 years) women). In stratified analyses by age, the variable for combining menopausal status and hormone therapy use was only included in the models for older women. When testing the effect of breastfeeding by both race and age, we combined two categories (6–11, ≥12 months) of longer duration of breastfeeding into one (≥6 months) to avoid having groups with small numbers of study participants after stratification.

As the commonly used definition (ER+ and/or PR+ plus HER2–) of the luminal A-like subtype probably includes both luminal B-like and luminal A-like tumors, we also used the 13^th^ St. Gallen International Breast Cancer Conference (2013) Expert Panel recommendation to define ER+/PR+/HER2– as the luminal A-like subtype [46], and repeated our analysis by the following five subtypes: luminal A-like (ER+/PR+/HER2–), luminal B-like-HER2– (ER+ or PR+ plus HER2–), luminal B-like-HER2+ (ER+ and/or PR+ plus HER2+), HER2-enriched (ER–/PR–/HER2+), and TNBC (ER–/PR–/HER2–). It is noteworthy that the St. Gallen Panel recommendation requires information on Ki-67 and percentage of PR in PR+ tumors; however, we lacked data on Ki-67 in all source studies and did not have quantitative data for PR in the Women’s LIFE Study. Moreover, in order to exclude the possibility that ORs for some reproductive factors associated with *in situ* breast cancer are different from those associated with the results presented here, we repeated our analyses after excluding all *in situ* breast cancer cases.

In reporting the results of trend tests or homogeneity tests, we considered a two-sided *P* value <0.05 as statistically significant. All analyses were performed using the SAS statistical package (Version 9.3, SAS Institute, Cary, NC, USA).

## Results

### Characteristics of case participants and controls

The subtypes of breast cancer in the 2658 case participants were distributed as: TNBC (*n* = 554, 20.8%), luminal A-like (*n* = 1517, 57.1%), luminal B-like (*n* = 360, 13.5%), and HER2-enriched (*n* = 227, 8.5%) (Table 1). The 2448 control participants comprised 1549 white women and 899 African-American women. Overall, mean age at menarche in control participants was 12.4 years; 81.3% of control participants had at least one completed pregnancy. Among control participants, the mean number of completed pregnancies was 2.7 in parous women, and their mean age at first completed pregnancy was 23.2 years. Among parous control participants who had ever breastfed (62.4%), the mean duration of breastfeeding was 12.1 months.

**Table 1:** Characteristics of breast cancer case participants and control participants

	Overall	By study		
		Women’s CARE	Women’s BCIS	Women’s LIFE
Case participants	*N* = 2658	*N* = 1197	*N* = 342	*N* = 1119
Mean age at diagnosis, years (SD, range)	46.7 (8.1, 22-64)	49.0 (8.6, 35-64)	51.8 (7.3, 35-64)	42.7 (5.4, 22-49)
Race				
White	1960 (73.7%)	678 (56.6%)	287 (83.9%)	995 (88.9%)
African-American	698 (26.3%)	519 (43.4%)	55 (16.1%)	124 (11.1%)
Subtype of breast cancer				
Triple-negative	554 (20.8%)	335 (28.0%)	21 (6.1%)	198 (17.7%)
Luminal A-like	1517 (57.1%)	645 (53.9%)	233 (68.1%)	639 (57.1%)
Luminal B-like	360 (13.5%)	121 (10.1%)	49 (14.3%)	190 (17.0%)
HER2-enriched	227 (8.5%)	96 (8.0%)	39 (11.4%)	92 (8.2%)
Control participants	*N* = 2448	*N* = 2011^a^	─	*N* = 437
Mean age at reference date, years (SD, range)	47.8 (8.3, 24-64)	48.9 (8.4, 35-64)	─	42.6 (4.9, 24-49)
Race				
White	1549 (63.3%)	1147 (57.0%)	─	402 (92.0%)
African-American	899 (36.7%)	864 (43.0%)	─	35 (8.0%)
Mean age at menarche, years (SD)	12.4 (1.6)	12.4 (1.6)	─	12.7 (1.5)
Ever had a completed (>26-week) pregnancy	1990 (81.3%)	1677 (83.4%)	─	313 (71.6%)
Among parous women	*N* = 1990	*N* = 1677		*N* = 313
Mean number of completed pregnancies (SD)	2.7 (1.5)	2.8 (1.6)	─	2.2 (1.1)
Mean age at first completed pregnancy (SD)	23.2 (5.7)	22.5 (5.3)	─	27.1 (6.2)
Ever breastfed	1242 (62.4%)	967 (57.7%)	─	275 (87.9%)
Mean duration of breastfeeding among those who breastfed, months (SD)	12.1 (15.0)	10.9 (14.4)	─	16.2 (16.5)

^a^Including those who also served as controls in the Women’s BCIS. Triple-negative = estrogen receptor (ER)–/progesterone receptor (PR)–/human epidermal growth factor receptor-2 (HER2)–, Luminal A-like = ER+ and/or PR+ plus HER2–, Luminal B-like = ER+ and/or PR+ plus HER2+, HER2-enriched = ER–/ PR–/HER2+. *CARE* Contraceptive and Reproductive Experiences Study, *BCIS* Breast Carcinoma *in situ* Study, *LIFE* Learning the Influence of Family and Environment Study

### Reproductive factors and risk of four ER/PR/HER2-defined subtypes

Age at menarche was not associated with TNBC (*P*_trend_ = 0.55; Table 2), luminal B-like (*P*_trend_ = 0.70), or HER2-enriched (*P*_trend_ = 0.56) breast cancer, but was associated with luminal A-like subtype (*P*_trend_ = 0.009). Women whose menarche occurred at age 14 years or later had a 23% lower risk of luminal A-like breast cancer (OR = 0.77, 95% CI = 0.64–0.92) than women whose menarche occurred at age 12 years or earlier. Number of completed pregnancies was not associated with the risk of TNBC (*P*_trend_ = 0.26), but was inversely associated with the risk of the other three subtypes (all *P*_*trend*_ ≤0.02).

**Table 2:** Multivariable adjusted odds ratio (OR) and 95% confidence interval (CI) for ER/PR/HER2-defined subtypes of breast cancer associated with reproductive factors

	Controls	Triple-negative		Luminal A-like		Luminal B-like		HER2-enriched	
	*N*	*N*	OR (95% CI)	*N*	OR (95% CI)	*N*	OR (95% CI)	*N*	OR (95% CI)
All women									
Age at menarche, years									
≤ 12	1292	289	1.00	820	1.00	186	1.00	110	1.00
13	634	158	1.13 (0.90–1.41)	419	0.98 (0.84–1.16)	99	1.06 (0.81–1.40)	66	1.22 (0.88–1.69)
≥ 14	522	107	0.89 (0.69–1.14)	278	0.77 (0.64–0.92)	75	0.92 (0.68–1.24)	51	1.07 (0.75–1.53)
Trend *P* value			*0.55*		*0.009*		*0.70*		*0.56*
*P* value for homogeneity of trends			*0.19*						
Number of completed pregnancies									
Never pregnant	250	68	1.00	224	1.00	56	1.00	31	1.00
Any completed pregnancies	1990	431	0.91 (0.67–1.24)	1104	0.79 (0.64–0.98)	263	0.82 (0.58–1.15)	176	0.78 (0.50–1.20)
1	399	98	0.95 (0.66–1.36)	277	0.92 (0.71–1.19)	84	1.17 (0.79–1.74)	50	1.02 (0.62–1.67)
2	709	168	0.98 (0.70–1.37)	438	0.81 (0.64–1.02)	109	0.88 (0.61–1.29)	59	0.69 (0.42–1.11)
≥3	882	165	0.84 (0.60–1.19)	389	0.64 (0.50–0.81)	70	0.53 (0.35–0.80)	67	0.65 (0.40–1.05)
Trend *P* value			*0.26*		*<0.0001*		*<0.0001*		*0.02*
*P* value for homogeneity of trends			*0.09*						
Only non-completed pregnancy	208	55	0.91 (0.60–1.38)	189	1.02 (0.76–1.35)	41	0.88 (0.55–1.39)	20	0.69 (0.38–1.26)
Parous women only^a,b^									
Age at first completed pregnancy, years									
≤20	799	165	1.00	297	1.00	69	1.00	62	1.00
21–24	510	107	1.03 (0.78–1.38)	274	1.22 (0.98–1.52)	63	1.18 (0.80–1.73)	43	1.05 (0.68–1.62)
25–29	348	85	1.08 (0.77–1.52)	275	1.64 (1.28–2.10)	56	1.22 (0.78–1.89)	33	1.10 (0.66–1.85)
≥30	333	73	0.80 (0.54–1.20)	256	1.17 (0.88–1.55)	75	1.09 (0.68–1.74)	38	0.96 (0.55–1.70)
Trend *P* value			*0.50*		*0.05*		*0.57*		*0.95*
*P* value for homogeneity of trends			*0.24*						
Duration of breastfeeding, months									
Never	748	166	1.00	370	1.00	81	1.00	60	1.00
Ever	1242	264	0.80 (0.63–1.02)	732	0.78 (0.65–0.94)	182	0.89 (0.65–1.23)	116	0.91 (0.63–1.32)
< 6	541	134	0.96 (0.74–1.26)	303	0.83 (0.68–1.02)	82	0.99 (0.70–1.41)	35	0.68 (0.43–1.07)
6–11	263	40	0.55 (0.37–0.82)	165	0.76 (0.59–0.99)	35	0.70 (0.44–1.12)	33	1.28 (0.78–2.09)
≥ 12	438	90	0.69 (0.50–0.96)	264	0.71 (0.56–0.90)	65	0.85 (0.56–1.30)	48	1.10 (0.69–1.75)
Trend *P* value			*0.006*		*0.004*		*0.28*		*0.36*
*P* value for homogeneity of trends			*0.08*						

Models in multivariate analysis included sub-study (the Women’s CARE Study or the Women’s BCIS Study, the Women’s LIFE Study), study site (Los Angeles, Detroit), race (white, African-American), reference age (in 5-year age categories), education (≤ high school, technical school or some college, college graduate), first-degree breast cancer family history (no, yes), body mass index (<25, 25–29, ≥30 kg/m^2^), a variable combining menopausal status and hormone therapy use (premenopausal; postmenopausal: never used hormone therapy, ever used hormone therapy; unknown menopausal status), lifetime recreational physical activity (inactive, ≤2.2, 2.3–6.6, 6.7–15.1, ≥15.2 annual metabolic equivalents of energy expenditure, hour/week), alcohol intake (never, former, current), cigarette smoking status (never, former, current), age at menarche (≤12,13, ≥14 years), number of completed pregnancies (never pregnant, 1, 2, ≥3, only non-completed pregnancy), oral contraceptive use (never, <1, 1–4, 5–9, ≥10 years). ^a^Models additionally included age at first completed pregnancy (≤20, 21–24, 25–29, ≥30 years) and duration of breastfeeding (never, <6, 6–11, ≥12 months). ^b^Additionally seven parous case participants who had missing information on breastfeeding were excluded. Triple negative = estrogen receptor (ER)–/progesterone receptor (PR)–/human epidermal growth factor receptor-2 (HER2)–, Luminal A-like = ER+ and/or PR+ plus HER2–, Luminal B-like = ER+ and/or PR+ plus HER2+, HER2-enriched = ER–/PR– /HER2 +

Among parous women, older age at first completed pregnancy was not associated with any ER/PR/HER2-defined specific subtypes, except for the luminal A-like subtype (*P*_trend_ = 0.05). There was a statistically significant inverse association between longer duration of breastfeeding and TNBC (*P*_trend_ = 0.006) and luminal A-like cancer (*P*_trend_ = 0.004), but no association with the other two subtypes of breast cancer (both *P*_trend_ ≥0.28).

None of the differences in trends across the four subtypes of breast cancer was statistically significant for age at menarche (*P* for homogeneity of trends = 0.19), parity (*P* for homogeneity of trends = 0.09), age at first completed pregnancy (*P* for homogeneity of trends = 0.24), or duration of breastfeeding (*P* for homogeneity of trends = 0.08).

### Reproductive factors and risk of TNBC and luminal A-like subtype by race

Among either white women or African-American women, risk of TNBC was not associated with age at menarche number of completed pregnancies, or age at first completed pregnancy (Table 3). However, among white women, risk of luminal A-like breast cancer was inversely associated with age at menarche (*P*_trend_ = 0.02) and number of completed pregnancies (*P*_trend_ = 0.0005).

**Table 3:** Multivariable adjusted odds ratio (OR) and 95% confidence interval (CI) for ER/PR/HER2-defined subtypes of breast cancer associated with reproductive factors by race

	White women					African-American women				
	Controls	Triple-negative		Luminal A-like		Controls	Triple-negative		Luminal A-like	
	*N*	*N*	OR (95% CI)	*N*	OR (95% CI)	*N*	*N*	OR (95% CI)	*N*	OR (95% CI)
All women										
Age at menarche, years										
≤12	793	167	1.00	631	1.00	499	122	1.00	189	1.00
13	433	102	1.12 (0.84–1.48)	327	0.90 (0.74–1.08)	201	56	1.09 (0.75–1.58)	92	1.18 (0.86–1.61)
≥14	323	74	0.99 (0.72–1.36)	220	0.78 (0.63–0.97)	199	33	0.69 (0.44–1.06)	58	0.75 (0.53–1.08)
Trend *P* value			*0.89*		*0.02*			*0.16*		*0.26*
*P* value for homogeneity of trends				*0.10*					*0.68*	
Number of completed pregnancies										
Never pregnant	199	56	1.00	200	1.00	51	12	1.00	24	1.00
Any completed pregnancies	1213	246	0.87 (0.61–1.24)	822	0.77 (0.61–0.97)	777	185	1.18 (0.59–2.36)	282	0.89 (0.51–1.54)
1	240	58	0.91 (0.59–1.40)	199	0.86 (0.65–1.16)	159	40	1.14 (0.54–2.43)	78	1.09 (0.60–1.97)
2	468	112	0.99 (0.67–1.45)	358	0.82 (0.63–1.06)	241	56	1.12 (0.54–2.35)	80	0.79 (0.44–1.42)
≥3	505	76	0.69 (0.45–1.05)	265	0.60 (0.45–0.79)	377	89	1.24 (0.60–2.57)	124	0.76 (0.43–1.36)
Trend *P* value			*0.11*		*0.0005*			*0.62*		*0.13*
*P* value for homogeneity of trends				*0.48*					*0.12*	
Only non-completed pregnancy	137	41	1.02(0.63–1.64)	156	1.08 (0.78–1.49)	71	14	0.86 (0.35–2.08)	33	0.97 (0.49–1.90)
Parous women only^a,b^										
Age at first completed pregnancy, years										
≤20	338	60	1.00	162	1.00	461	105	1.00	135	1.00
21–24	347	66	1.03 (0.68–1.56)	199	1.09 (0.82–1.44)	163	41	1.01 (0.65–1.58)	75	1.40 (0.96–2.04)
25–29	257	64	1.09 (0.69–1.70)	227	1.55 (1.14–2.10)	91	21	1.05 (0.59–1.89)	48	1.75 (1.10–2.80)
≥30	271	55	0.70 (0.42–1.17)	232	1.13 (0.80–1.60)	62	18	1.21 (0.61–2.41)	24	0.95 (0.51–1.77)
Trend *P* value			*0.21*		*0.10*			*0.62*		*0.24*
*P* value for homogeneity of trends				*0.02*					*0.66*	
Duration of breastfeeding, months										
Never	368	66	1.00	228	1.00	380	100	1.00	142	1.00
Ever	845	179	0.97 (0.68–1.38)	592	0.81 (0.64–1.02)	397	85	0.67 (0.47–0.96)	140	0.78 (0.57–1.06)
< 6	343	84	1.14 (0.78–1.68)	230	0.85 (0.66–1.10)	198	50	0.79 (0.52–1.19)	73	0.82 (0.57–1.18)
6–11	194	29	0.62 (0.37–1.05)	134	0.75 (0.54–1.02)	69	11	0.54 (0.26–1.09)	31	1.06 (0.63–1.77)
≥ 12	308	66	0.88 (0.56–1.39)	228	0.77 (0.57–1.04)	130	24	0.55 (0.32–0.94)	36	0.57 (0.36–0.90)
Trend *P* value			*0.27*		*0.07*			*0.01*		*0.04*
*P* value for homogeneity of trends				*0.94*					*0.47*	

Triple negative = estrogen receptor (ER)–/progesterone receptor (PR)–/human epidermal growth factor receptor-2 (HER2)–, Luminal A-like = ER+ and/or PR+ plus HER2. Models in multivariate analysis included sub-study (the Women’s CARE Study or the Women’s BCIS Study, the Women’s LIFE Study), study site (Los Angeles, Detroit), reference age (in 5-year age categories), education (≤ high school, technical school or some college, college graduate), first-degree breast cancer family history (no, yes), body mass index (<25, 25–29, ≥30 kg/m^2^), a variable combining menopausal status and hormone therapy use (premenopausal; postmenopausal: never used hormone therapy, ever used hormone therapy; unknown menopausal status), lifetime recreational physical activity (inactive, ≤2.2, 2.3–6.6, 6.7–15.1, ≥15.2 annual metabolic equivalents of energy expenditure, hour/week), alcohol intake (never, former, current), cigarette smoking status (never, former, current), age at menarche (≤12,13, ≥14 years), number of completed pregnancies (never pregnant, 1, 2, ≥3, only non-completed pregnancy), oral contraceptive use (never, <1, 1–4, 5–9, ≥10 years). ^a^Models additionally included age at first completed pregnancy (≤20, 21–24, 25–29, ≥30 years) and duration of breastfeeding (never, <6, 6–11, ≥12 months). ^b^Additionally, three white parous cases who had missing information on breastfeeding were excluded

Longer duration of breastfeeding was modestly associated with a lower risk of TNBC in parous African-American women and a lower risk of the luminal A-like subtype in both parous white women and parous African-American women; however, statistically significant dose-response relationships were only observed in TNBC (*P*_trend_ = 0.01) and the luminal A-like subtype (*P*_trend_ = 0.04) among the African-American women. Among parous African-American women, breastfeeding for 12 months or longer compared to never breastfeeding was associated with a 45% lower risk of TNBC (OR = 0.55, 95% CI = 0.32–0.94), and 43% lower risk of luminal A-like breast cancer (OR = 0.57, 95% CI = 0.36–0.90).

The difference in trends across TNBC and luminal A-like breast cancer was only statistically significant for age at first completed pregnancy among parous white women (*P* for homogeneity of trends = 0.02); none of the other factors differed significantly between these two subtypes (*P* for homogeneity of trends ≥0.10).

### Reproductive factors and risk of TNBC and the luminal A-like subtype by age

Similar to the results for all age groups combined, early age at menarche, nulliparity, and older age at first completed pregnancy were not associated with risk of TNBC in either younger or older women (*P*_trend_ ≥0.13, Table 4). However, younger women (aged 20–44 years) whose menarche occurred at age 14 years or later had a 37% lower risk of luminal A-like breast cancer (OR = 0.63, 95% CI = 0.47–0.85) than those whose menarche occurred at age 12 years or earlier (*P*_trend_ = 0.004). Number of completed pregnancies was inversely associated with the risk of luminal A-like subtype cancer among both younger (*P*_trend_ = 0.02) and older women (*P*_trend_ = 0.006). Among older parous women, age at first completed pregnancy was positively associated with the risk of the luminal A-like subtype (*P*_trend_ = 0.01); those who had their first completed pregnancies at or after age 30 years had a higher risk of the luminal A-like subtype than those who had their first completed pregnancies at or prior to age 20 years (OR = 1.32, 95% CI = 0.91–1.90). Duration of breastfeeding was inversely associated with the risk of both TNBC and luminal A-like breast cancer subtypes in both younger and older parous women.

**Table 4:** Multivariable adjusted odds ratio (OR) and 95% confidence interval (CI) for ER/PR/HER2-defined subtypes of breast cancer associated with reproductive factors by age

	Younger (20–44 years)					Older^a^ (45–64 years)				
	Controls	Triple-negative		Luminal A-like		Controls	Triple-negative		Luminal A-like	
	*N*	*N*	OR (95% CI)	*N*	OR (95% CI)	*N*	*N*	OR (95% CI)	*N*	OR (95% CI)
All women										
Age at menarche, years										
≤12	491	139	1.00	299	1.00	801	150	1.00	521	1.00
13	252	94	1.35 (0.98–1.85)	152	0.94 (0.72–1.22)	382	64	0.90 (0.65–1.25)	267	0.94 (0.77–1.16)
≥14	227	58	0.86 (0.60–1.23)	98	0.63 (0.47–0.85)	295	49	0.85 (0.59–1.22)	180	0.85 (0.67–1.08)
Trend *P* value			*0.72*		*0.004*			*0.33*		*0.18*
*P*-value for homogeneity of trends				*0.06*					*0.92*	
Number of completed pregnancies										
Never pregnant	115	47	1.00	91	1.00	135	21	1.00	133	1.00
Any completed pregnancies	735	207	0.88 (0.58–1.32)	368	0.75 (0.54–1.05)	1255	224	1.06 (0.64–1.77)	736	0.82 (0.62–1.09)
1	193	48	0.75 (0.46–1.23)	107	0.83 (0.56–1.23)	206	50	1.30 (0.73–2.31)	170	1.01 (0.72–1.42)
2	289	83	0.94 (0.60–1.48)	159	0.82 (0.57–1.19)	420	85	1.15 (0.67–1.96)	279	0.80 (0.59–1.09)
≥ 3	253	76	0.98 (0.61–1.56)	102	0.59 (0.40–0.88)	629	89	0.85 (0.49–1.48)	287	0.70 (0.51–0.96)
Trend *P* value			*0.73*		*0.02*			*0.13*		*0.006*
*P* value for homogeneity of trends				*0.03*					*0.83*	
Only non-completed pregnancy	120	37	0.91 (0.54–1.53)	90	1.03 (0.68–1.57)	88	18	1.08 (0.54–2.19)	99	1.10 (0.74–1.65)
Parous women only^b,c^										
Age at first completed pregnancy, years										
≤ 20	235	71	1.00	85	1.00	564	94	1.00	212	1.00
21–24	142	43	1.02 (0.63–1.65)	80	1.44 (0.95–2.17)	368	64	1.05 (0.72–1.54)	194	1.11 (0.85–1.44)
25–29	168	50	0.94 (0.57–1.56)	99	1.48 (0.97–2.27)	180	35	1.20 (0.74–1.94)	176	1.84 (1.34–2.51)
≥30	190	42	0.62 (0.34–1.12)	102	1.15 (0.71–1.86)	143	31	1.08 (0.61–1.89)	154	1.32 (0.91–1.90)
Trend *P* value			*0.15*		*0.52*			*0.60*		*0.01*
*P* value for homogeneity of trends				*0.07*					*0.27*	
Duration of breastfeeding, months										
Never	204	64	1.00	100	1.00	544	102	1.00	270	1.00
Ever	531	142	0.75 (0.50–1.12)	266	0.70 (0.50–0.99)	711	122	0.85 (0.62–1.17)	466	0.83 (0.67–1.03)
< 6	209	69	0.93 (0.60–1.44)	97	0.72 (0.49–1.05)	332	65	0.97 (0.67–1.39)	206	0.90 (0.70–1.16)
6–11	126	22	0.49 (0.27–0.89)	64	0.68 (0.44–1.07)	137	18	0.62 (0.35–1.10)	101	0.84 (0.60–1.17)
≥ 12	196	51	0.60 (0.35–1.01)	105	0.68 (0.44–1.05)	242	39	0.79 (0.50–1.24)	159	0.71 (0.53–0.96)
Trend *P* value			*0.02*		*0.12*			*0.17*		*0.03*
*P* value for homogeneity of trends				*0.32*					*0.93*	

Triple-negative = estrogen receptor (ER)–/progesterone receptor (PR)–/human epidermal growth factor receptor-2 (HER2)–, Luminal A-like = ER+ and/or PR+ plus HER2. Models in multivariate analysis included sub-study (the Women’s CARE Study or the Women’s BCIS Study, the Women’s LIFE Study), study site (Los Angeles, Detroit), race (white, Africa-American), reference age (in 5-year age categories), education (≤ high school, technical school or some college, college graduate), first-degree breast cancer family history (no, yes), body mass index (<25, 25–29, ≥30 kg/m^2^), lifetime recreational physical activity (inactive, ≤2.2, 2.3–6.6, 6.7–15.1, ≥15.2 annual metabolic energy equivalents, hour/week), alcohol intake (never, former, current), cigarette smoking status (never, former, current), age at menarche (≤12,13, ≥14 years), number of completed pregnancies (never pregnant, 1, 2, ≥3, only non-completed pregnancy), oral contraceptive use (never, <1, 1–4, 5–9, ≥10 years). ^a^Additionally adjusted for a variable combining menopausal status and hormone therapy use (premenopausal; postmenopausal: never used hormone therapy, ever used hormone therapy; unknown menopausal status). ^b^Models additionally included age at first completed pregnancy (≤20, 21–24, 25–29, ≥30 years) and duration of breastfeeding (never, <6, 6–11, ≥12 months). ^c^Additionally three younger case participants who had missing information on breastfeeding were excluded

The differences in trends across TNBC and luminal A-like breast cancer were statistically significant only for the number of completed pregnancies among younger women (*P* for homogeneity of trends = 0.03).

### Breastfeeding and risk of TNBC and luminal A-like breast cancer by race and age

In our stratified analyses examining the association between breastfeeding and TNBC or luminal A-like breast cancer by both race and age, we observed a stronger protective effect of longer duration of breastfeeding against the risk of TNBC among younger parous African-American women (*P*_trend_ *=* 0.0004, Table 5); those who breastfed for 6 months or longer had an 82% lower risk of TNBC compared to their counterparts who had never breastfed (OR = 0.18, 95% CI = 0.07–0.46). The other ORs for the association between breastfeeding for 6 months or longer and TNBC or the luminal A-like subtype were <1, but their corresponding 95% CIs included the null value.

**Table 5:** Multivariable adjusted odds ratio (OR) and 95% confidence interval (CI) for ER/PR/HER2-defined subtypes of breast cancer associated with duration of breastfeeding among parous women by race and age

	White women					African-American women				
	Controls	Triple-negative		Luminal A-like		Controls	Triple-negative		Luminal A-like	
	*N*	*N*	OR (95% CI)	*N*	OR (95% CI)	*N*	*N*	OR (95% CI)	*N*	OR (95% CI)
Younger (20–44 years)^a^										
Duration of breastfeeding, months										
Never	85	27	1.00	57	1.00	119	37	1.00	43	1.00
Ever	383	105	0.85 (0.49–1.49)	218	0.71 (0.46–1.11)	148	37	0.43 (0.22–0.83)	48	0.58 (0.32–1.06)
<6	136	43	0.94 (0.52–1.72)	72	0.70 (0.43–1.14)	73	26	0.67 (0.33–1.36)	25	0.64 (0.32–1.26)
≥6	247	62	0.76 (0.41–1.41)	146	0.73 (0.45–1.18)	75	11	0.18 (0.07–0.46)	23	0.50 (0.24–1.06)
Trend *P* value			*0.34*		*0.28*			*0.0004*		*0.07*
*P* value for homogeneity of trends				*0.93*					*0.09*	
Older (45–64 years)^b^										
Duration of breastfeeding, months										
Never	283	39	1.00	171	1.00	261	63	1.00	96	1.00
Ever	462	74	1.06 (0.65–1.72)	374	0.83 (0.63–1.10)	249	48	0.74 (0.47–1.17)	92	0.90 (0.62–1.30)
< 6	207	41	1.30 (0.78–2.19)	158	0.91 (0.67–1.25)	125	24	0.69 (0.39–1.21)	48	0.94 (0.60–1.46)
≥ 6	255	33	0.75 (0.41–1.38)	216	0.73 (0.53–1.02)	124	24	0.81 (0.46–1.43)	44	0.85 (0.54–1.35)
Trend *P* value			*0.41*		*0.07*			*0.35*		*0.49*
*P* value for homogeneity of trends				*0.85*					*0.72*	

Triple negative = estrogen receptor (ER)–/progesterone receptor (PR)–/human epidermal growth factor receptor-2 (HER2)–, Luminal A-like = ER+ and/or PR+ plus HER2. Models in multivariate analysis included sub-study (the Women’s CARE Study or Women’s BCIS Study, Women’s LIFE Study), study site (Los Angeles, Detroit), reference age (in 5-year age categories), education (< high school, technical school or some college, college graduate), first-degree breast cancer family history (no, yes), body mass index (<25, 25–29, ≥30 kg/m^2^), lifetime recreational physical activity (inactive, ≤2.2, 2.3–6.6, 6.7–15.1, ≥15.2 annual metabolic energy equivalents, hour/week), alcohol intake (never, former, current), cigarette smoking status (never, former, current), age at menarche (≤12,13, ≥14 years), number of completed pregnancies (1, 2, ≥3), oral contraceptive use (never, <1, 1–4, 5–9, ≥10 years) and age at first completed pregnancy (≤20, 21–24, 25–29, ≥30 years). ^a^Additionally three white younger case participants who had missing information on breastfeeding were excluded. ^b^Additionally adjusted for a variable combining menopausal status and hormone therapy use (premenopausal; postmenopausal: never used hormone therapy, ever used hormone therapy; unknown menopausal status)

### Additional results

The results for ER+/PR+/HER2– breast cancer, the modified definition of the luminal A-like subtype recommended by the 13^th^ St. Gallen International Breast Cancer Conference (2013) Expert Panel, were similar to data presented in the tables for the more commonly used definition (ER+ and/or PR+ plus HER2–) of the luminal A-like breast cancer subtype. Moreover, when we excluded women diagnosed with *in situ* breast cancer, we found that the results for TNBC were unchanged.

## Discussion

In our pooled analysis of data from three population-based case-control studies of women aged 20–64 years, we found that longer duration of breastfeeding was associated with decreased risk of both TNBC and the luminal A-like subtype, especially for TNBC among younger (20–44 years) parous African-American women. Younger parous African-American women who breastfed for 6 months or longer had an 82% lower risk of TNBC than their counterparts who had never breastfed. If this is verified in future research, promotion of breastfeeding, especially in younger African-American women may have a particularly strong impact given the higher risk of TNBC among African-American women [13–16].

Breastfeeding has been proposed to protect against breast cancer through hormonal mechanisms that include postponing the resumption of ovulatory menstrual cycles after a pregnancy [47], reducing estrogen levels in the breast [48], and having fully differentiated breast tissue that is less susceptible to the hormone milieu [49]. It has also been proposed that breastfeeding has a direct mechanical effect, by which carcinogenic agents are excreted from the breast ductal tissue [50]. Moreover, as the amount of involution of terminal duct lobular units has been inversely associated with risk of breast cancer [51], it has been hypothesized that long-term breastfeeding could promote such involution, resulting in decreased risk of breast cancer, and particularly basal-like breast cancers [52, 53]. The basal-like subtype is defined according to cDNA microarray technology [54]. Because cDNA microarray technology is not yet available clinically, TNBC has become a commonly used proxy for the basal-like subtype in clinical and epidemiologic studies, despite the fact that TNBC and the basal-like subtype are discordant in 20–30% of cases [17, 55].

Our finding of a protective effect of a longer duration of breastfeeding against the risk of TNBC agrees with results from six previous case-control studies [6, 22, 23, 25, 27, 32], a pooled analysis of two case-control studies [8], and one prospective study [21]. Moreover, three case-control studies [15, 26, 28] and the African-American Breast Cancer Epidemiology and Risk (AMBER) Consortium [31] report a non-statistically significant protective effect of breastfeeding on TNBC (risk estimates for the highest category of breastfeeding versus never breastfeeding or lowest category = 0.70–0.91). No inverse association was observed in one case-control study [24] and one prospective study [30]. A meta-analysis published in 2015 [56] concluded that both case-control studies and cohort studies support the evidence that ever breastfeeding is associated with decreased risk of ER–/PR– breast cancer and of TNBC. On average, ever breastfeeding was associated with a 10% decrease in the risk of ER–/PR– breast cancer and up to a 20% reduction in the risk of TNBC. A more recent meta-analysis published in 2016 [57] showed that ever breastfeeding was associated with a 21% decrease in the risk of TNBC (summary OR = 0.79, 95% CI = 0.66–0.94). However, neither of these meta-analyses provided data on the association between breastfeeding duration and risk of TNBC.

Longer duration of breastfeeding was also associated with decreased risk of the luminal A-like subtype in our pooled analysis. Among previous studies that reported results on the association between breastfeeding and the luminal A-like subtype, breastfeeding was associated with decreased risk of the luminal A-like subtype in three case-control studies [6, 15, 22], but was not associated with risk of this subtype in three other studies [25, 27, 30]. The meta-analyses published in 2015 [56] and in 2016 [57] did not provide summary results specifically for the luminal A-like subtype. However, the earlier publication [56] provided summary results for the ER+/PR+ subtype and concluded that the evidence for an inverse association between ever breastfeeding and ER+/PR+ breast cancer was observed in case-control studies (summary OR = 0.86, 95% CI = 0.79–0.92), but not in cohort studies (summary RR = 1.00, 95% CI = 0.90–1.10); the later one [57] provided results for the luminal subtype (ER+ and/or PR+) and showed that ever breastfeeding was associated with a 23% decrease in risk of the luminal subtype. In summary, the evidence for a protective effect of breastfeeding against the risk of luminal A-like cancer is not as strong as that for TNBC.

It has been well-documented that estrogen and progesterone play important roles in breast tumorigenesis [58–60], and their effects on breast cells are mediated by their respective receptors, the ER and the PR [61–64]. Furthermore, gene expression studies using cDNA microarray technology show that the luminal-A subtype is associated with ER signaling, whereas TNBC is characterized by a basal-like molecular profile, typically expressing genes involved in cell proliferation and differentiation [54, 65]. In our pooled analysis, the fact that early age at menarche, nulliparity, and late age at first completed pregnancy were all associated with increased risk of luminal A-like breast cancer, but not with TNBC, provides additional evidence that these three reproductive factors affect breast cancer risk predominantly through hormonal mechanisms.

Previous epidemiologic data on the impact of these three factors on the risk of luminal A-like cancer and TNBC are inconsistent, particularly for TNBC. Early age at menarche was not associated with risk of TNBC in three case-control studies [22, 23, 27], three prospective studies [20, 21, 30], and one pooled analysis of 34 studies [9]; however, it was a risk factor for TNBC in several other studies [10, 15, 24–26, 29]. Some studies found no association between nulliparity and risk of TNBC [6, 9, 10, 26, 27, 30, 57], whereas another reported an increased risk of TNBC associated with nulliparity [23]. Others showed a reduced risk of TNBC with nulliparity [20, 25, 28, 31, 32]. Some studies found no association of risk of TNBC with late age at first full-term pregnancy [6, 10, 20] or first birth [21, 26, 27, 29, 30, 57]; one study showed an increased risk of TNBC associated with late age at first birth [24] whereas others showed a reduced risk of TNBC associated with increasing age at first birth [9, 25]. Our findings are consistent with the majority of previous analyses of early age at menarche [9, 20–23, 27, 30], nulliparity [6, 9, 10, 26, 27, 30, 57], and late age at first full-term pregnancy [6, 10, 20] or first birth [21, 26, 27, 29, 30, 57], showing that these three reproductive factors were not associated with the risk of TNBC.

African-American women are more likely than white women to be diagnosed with TNBC, especially at a young age (<45 years) [13–16]. In the high risk group, we observed an inverse association between breastfeeding and risk of TNBC, suggesting that the protective effect of breastfeeding against risk of TNBC is modified by age and race. To our knowledge, no data have previously been published on effect modification by age for the association between breastfeeding and risk of TNBC. The observed reductions in risk of breast cancer overall in many previously published studies have been stronger for or restricted to younger or premenopausal women [66–71]; however, in some studies, this reduction in risk of breast cancer risk was observed among postmenopausal women [72–74] or was negligible in both premenopausal and postmenopausal women [75, 76]. Two studies found that the protective effect of breastfeeding against breast cancer decreased with the increasing time since last pregnancy [66, 77]. The reduction in risk of TNBC associated with breastfeeding was stronger for younger than for older African-American women, which was similar to the results for breast cancer overall. Moreover, there are few data on whether race modifies the association between breastfeeding and TNBC. The study of Work et al. provides some evidence of effect modification: multiparous women (≥3 live births) who never breastfed were at increased risk of ER–/PR– breast cancer, whereas multiparous women with a history of breastfeeding were at decreased risk of ER–/PR– breast cancer [32]. These associations were more apparent in African-American women than in non-Hispanic white women. Using data from the AMBER Consortium, Palmer et al. found that breastfeeding ameliorated the increased risk of TNBC associated with multiparity [31].

Our data also provide some evidence that breastfeeding may be important for limiting the increased risk of TNBC among parous African-American women, but not among parous white women (data not shown). The observed racial difference in our results for the inverse association between breastfeeding and risk of TNBC could not be explained by breastfeeding duration; African-American controls and case participants with TNBC had a shorter duration of breastfeeding on average than did white controls and case participants with TNBC (mean breastfeeding duration among those who ever had breastfed: 10.8 and 10.2 months for African-American controls and TNBC case participants, respectively; 12.7 and 13.1 months among white controls and TNBC case participants, respectively). The observed difference in breastfeeding results between African-American women and white women could be related to the differences in other characteristics, such as genetic susceptibility, and requires further study.

Strengths of this pooled analysis include its size, especially the large number of case participants with incident TNBC. Furthermore, the data used in this analysis were collected by trained interviewers, who administered standardized, in-person interviews using structured questionnaires, which were nearly identical across three source studies.

Several limitations of the current study must be considered. First, approximately 36% of our case participants had missing data on at least one of the receptors (ER, PR, or HER2). We compared our measures of reproductive factors in case patients with and without known ER, PR, or HER2. No statistically significant differences were detected for age at menarche, number of completed pregnancies, or duration of breastfeeding, whereas case patients with information on ER/PR/HER2 were 0.9 years older on average at first completed pregnancy than those with missing information on ER, PR, or HER2 (data not shown). The small difference in average age at first completed pregnancy is unlikely to have altered the observed associations differentially by tumor subtypes.

Second, ER/PR/HER2 status in two of our source studies [6, 35] was assayed at the same laboratory using the same methods, whereas in the third source study [33] the information on ER/PR/HER2 status was abstracted from medical records collected by the LACSP. Our previous validation study showed that the associations between reproductive factors and risk of the ER/PR subtypes of breast cancer were similar, whether the ER/PR values were from the same centralized laboratory at the USC providing assays for this analysis or from the LACSP [44]. In addition, we repeated our analyses with the two of our source studies with ER/PR/HER2 values from the centralized USC laboratory and found that the results were similar (data not shown) to those presented here.

A final limitation is that IHC was used to assess HER2 protein overexpression without validation by fluorescent *in situ* hybridization (FISH) analysis in the Women’s CARE Study and the Women’s BCIS Study. Based on previous validation results from the same centralized USC pathology laboratory, 7.4% of breast tumors with *HER2* gene amplification in FISH analysis were false negative by 10H8-IHC (scored as 0 or 1+) and 9.7% of breast cancers without *HER2* gene amplification in FISH analysis were false positive [42]. These misclassifications could cause bias towards the null for testing heterogeneity across subtypes involving HER2– versus HER2+ tumors, such as TNBC versus HER2-enriched cancer.

## Conclusions

In this pooled analysis, longer duration of breastfeeding was associated with decreased risk of TNBC especially in younger parous African-American women, suggesting a potential role of breastfeeding in prevention of TNBC among these women.

## Abbreviations

BCIS: breast carcinoma *in situ*
BMI: body mass index
CARE: Contraceptive and Reproductive Experiences
CIs: confidence intervals
ER: estrogen receptor
FISH: fluorescent *in situ* hybridization
HER2: human epidermal growth factor receptor-2
IHC: immunohistochemistry
LA: Los Angeles
LACSP: Los Angeles Cancer Surveillance Program
LCIS: lobular carcinoma *in situ*
LIFE: Learning the Influence of Family and Environment
MET: metabolic energy equivalent
NICHD: National Institute of Child Health and Human Development
ORs: odds ratios
PR: progesterone receptor
RR: relative risk
TNBC: triple-negative breast cancer
USC: University of Southern California
